# State LGBTQ policy environments and the cancer burden in sexual and gender minoritized communities in the United States

**DOI:** 10.1002/cam4.70097

**Published:** 2024-08-14

**Authors:** Ben C. D. Weideman, Donna McAlpine

**Affiliations:** ^1^ Division of Health Policy and Management School of Public Health, University of Minnesota Minneapolis Minnesota USA

**Keywords:** cancer, cancer survivorship, LGBTQ policy, sexual and gender minority

## Abstract

**Purpose:**

Our objective was to assess the association between state policies related to sexual orientation and gender identity (SOGI) and cancer prevalence and survivorship indicators in a sexual and gender minoritized (SGM) population in the United States.

**Methods:**

Data from the 2017–2021 Behavioral Risk Factor Surveillance System were used to measure cancer diagnosis, physical and mental health, and substance use for SGM adult cancer survivors. A state policy *Z*‐score, ranging from most restrictive to most protective state policies related to SOGI, was computed from data available from the Movement Advancement Project. Survey‐weighted logistic regression was used to test the relationship between state policies and cancer‐related outcomes for SGM people.

**Results:**

More protective state policies were associated with lower odds of a cancer diagnosis (adjusted odds ratio [AOR]: 0.92; 95% confidence interval [CI]: 0.87–0.97). Among SGM cancer survivors, increasing protective state policies were associated with lower odds of poor physical health (AOR: 0.83; 95% CI: 0.74–0.94), lower odds of difficulty walking or climbing stairs (AOR: 0.90; 95% CI: 0.80–1.00), and lower odds of difficulty concentrating or remembering (AOR: 0.87; 95% CI: 0.78–0.98). No significant associations were found between state policies and mental health, depression, substance use, diabetes, or cardiovascular disease among SGM cancer survivors.

**Conclusion:**

SGM people diagnosed with cancer are more likely to live in restrictive policy states, and survivors in those states have worse physical health and cognitive disability. Additional research should investigate potential causal relationships between state policies and SGM cancer outcomes.

## Abstract 2

There is evidence that SGM people living in states with restrictive LGBTQ policies are more likely to have cancer and these survivors have poorer physical health and cognitive disability. Protective LGBTQ social policies may be necessary to eliminate cancer disparities.

## 1 INTRODUCTION

Sexual and gender minoritized (SGM) populations, which include people who identify as lesbian, gay, bisexual, transgender, and/or queer (LGBTQ) and people who do not identify as cisgender and/or heterosexual,^1^ have a significant cancer burden.^2^ The size of the SGM population is growing^3^ and may now exceed 20 million in the United States.^4^ Given population growth and the fact that nearly all sexual orientation and gender identity (SOGI) data are under‐reports due to social stigma,^5^ a previous approximation of 0.5–1 million SGM cancer survivors in the United States^6^ is likely a substantial underestimate. There is a critical gap in SGM cancer research exacerbated by the absence of comprehensive SOGI surveillance data.^2^, ^7^, ^8^, ^9^, ^10^, ^11^, ^12^, ^13^, ^14^ Several prominent US research institutions have called for more efforts to address these gaps.^2^, ^9^, ^15^, ^16^, ^17^, ^18^

### 1.1 SGM cancer disparities

SGM people face inequities across the cancer continuum.^19^ SGM people are at a disproportionate risk for cancer. High rates of human papillomavirus (HPV) and human immunodeficiency virus (HIV) infections among SGM people,^20^, ^21^, ^22^, ^23^ associated with 15.4% of worldwide cancers,^24^ lead to higher rates of anal, cervical, and oropharyngeal, and other infection‐related cancers.^25^, ^26^, ^27^, ^28^ Higher rates of alcohol consumption, smoking, tanning, and stress, and fewer cancer screenings additionally contribute to the SGM cancer burden.^19^, ^29^, ^30^, ^31^, ^32^, ^33^, ^34^, ^35^ SGM people face additional challenges in cancer care, including negative experiences with providers, a lack of culturally tailored care, and discrimination.^19^, ^26^, ^36^, ^37^, ^38^, ^39^, ^40^ Cancer risks and inequities vary across subgroups. For example, transgender people have especially high HIV infection rates, increasing risk for certain cancers (e.g., anal cancer),^22^, ^26^, ^28^, ^41^ and cancer specialists may be particularly uncomfortable or incompetent in providing care to transgender cancer patients due in part to a lack of protocols.^42^

Studies of SGM cancer survivorship demonstrate several disparities in mental and physical health, substance use, and access to health care.^43^, ^44^ Relative to cisgender and heterosexual cancer survivors, SGM cancer survivors report worse mental health (anxiety, depression, and distress) and quality of life, and higher rates of smoking and alcohol use.^44^, ^45^, ^46^, ^47^, ^48^, ^49^, ^50^, ^51^ They also exhibit poorer physical health, less physical activity, and more comorbidities such as cardiovascular disease and diabetes.^44^, ^50^, ^51^, ^52^ SGM cancer survivorship trends differ in scope and magnitude across SGM subgroups,^50^ as well as by race/ethnicity, age, geography, and cancer type.^47^, ^50^, ^51^ Certain SGM subgroups (e.g., nonbinary people) and cancer types remain relatively understudied.^14^, ^44^ This evidence highlights the need for more research to address and eliminate SGM cancer disparities.

### 1.2 LGBTQ policy and health disparities

The relationship between policy and cancer in SGM communities has received little attention in cancer‐related research. Government policies directed at the SGM community can be protective (e.g., anti‐bullying laws) or restrictive (e.g., gender‐affirming care bans). Record numbers of restrictive anti‐LGBTQ policies have been introduced and passed in US state legislatures in recent years,^53^ curtailing the prior trend of increasing LGBTQ protections since 2010.^54^ US states now vary widely in terms of protective or restrictive LGBTQ policy environments, reflecting an increasingly polarized political climate.^55^

Multiple studies have linked state LGBTQ policies to SGM disparities in mental health, substance use, and access to care.^56^, ^57^, ^58^, ^59^, ^60^, ^61^, ^62^ For example, White and colleagues found poorer physical health, higher prevalence of depression, a greater number of mental disability days, and higher rates of smoking and alcohol use among sexually minoritized adults living in US states with restrictive policies compared to their counterparts living in states with more protective policies.^56^ Overall, these studies provide initial evidence that LGBTQ state policy environments matter for the health of SGM people and the fact that LGBTQ policies are associated with outcomes such as smoking and alcohol use suggests that they may also matter for cancer outcomes. However, to our knowledge there has not been a study that explores the relationship between state LGBTQ policies and SGM cancer prevalence or health outcomes among SGM cancer survivors.

Structural discrimination, which is reflected by and enacted through social policy, is central to the major theoretical models that guide SGM health disparities research. The prevailing framework, minority stress theory, posits that stigma and discrimination (both structural and interpersonal) elevate chronic and acute levels of stress in marginalized groups, which negatively affects health.^63^, ^64^ Minority stress has been shown to have biological effects in SGM individuals that may impact the incidence and experience of cancer.^65^ In concert, fundamental cause theory argues that structural stigmatizing mechanisms regulate access to resources (social, psychological, economic, health system, etc.) that disadvantage SGM individuals and cause health inequities to emerge and persist.^66^, ^67^ While the consensus in SGM health disparities research is that structural discrimination matters, most SGM cancer studies have focused on individual‐level variables (e.g., health behaviors)^11^, ^44^ when those variables alone may be insufficient for explaining the full scope of SGM cancer inequities. Socio‐structural approaches to the problem of cancer in SGM communities may also yield information about new strategies for intervention.

State policy environments are complex compositions of LGBTQ laws across social domains (health care, family, workplace, etc.) that vary in enforcement and change over time. The mechanisms by which LGBTQ policies may shape SGM cancer outcomes are also complex and varied: increasing minority stress, limiting social support, and financial toxicity to name a few. This study aims to further this nascent area of research by quantifying the relationship between state‐level policy environments on average and SGM cancer burden in terms of (1) cancer prevalence and (2) cancer survivorship outcomes. We hypothesize that states with more protective policy environments will produce better SGM cancer outcomes.

## 2 METHODS

### 2.1 Analytic sample

The study was exempt from approval by the University of Minnesota's Institutional Review Board due to being a secondary analysis of publicly available data. We combined 2017–2021 Behavioral Risk Factor Surveillance System (BRFSS) data from states that elected to include the optional SOGI module to collect data regarding SOGI. This resulted in data from 42 US states.

BRFSS partners with state health departments to administer over 400,000 telephone surveys annually, collecting data on health‐related risk factors, preventative service use, and chronic conditions in a non‐institutionalized adult population. We selected respondents who indicated an SGM status in BRFSS SOGI measures, including those with non‐heterosexual sexual orientation (lesbian or gay, bisexual, or something else), and/or a non‐cisgender gender modality (transgender, male‐to‐female; transgender, female‐to‐male; or transgender, gender nonconforming). Listwise deletion was used to handle missing data for cancer diagnosis and control variables, each of which had less than 1.6% missing data and low potential for bias. The exception to this approach was income, which had a substantially higher rate of missing responses (14.9%) that was included as a category in analyses following previous studies.^50^, ^52^ The final analytic sample included 56,309 SGM respondents.

### 2.2 Dependent variables

To estimate cancer prevalence, we used yes versus no responses to two questions: (1) “Have you ever been told that you have skin cancer?” and (2) “Have you ever been told that you have cancer, other than skin cancer?” Respondents answering “yes” to either question were considered cancer survivors.

Outcomes for cancer survivors consisted of BRFSS measures of physical and mental health and substance use. Poor mental health and poor physical health were measured by asking respondents to report the number of days in the last 30 days where, respectively, their mental and physical health was not good. BRFSS computes binary variables, which this study used, such that ≥14 days was considered to be poor mental or physical health. To capture physical and mental conditions, we used yes versus no responses to the question “have you ever been told you have (condition)?” Depression, diabetes, and cardiovascular disease were included because those conditions affect cancer survival and treatment and have been evaluated in previous studies of SGM cancer survivorship.^51^, ^52^ Three questions about heart attack, coronary heart disease, and stroke were combined for the measure of cardiovascular disease following a previous study.^52^

Since cancer survivors are at increased risk for long term functional limitations,^68^ we included a BRFSS item assessing physical disability as having serious difficulty walking or climbing up stairs (yes or no) and an item assessing cognitive disability as having serious difficulty concentrating, remembering, or making decisions because of a physical, mental, or emotional condition (yes or no). Lastly, since avoiding substance use is strongly recommended for cancer survivors,^69^ we included measures for current smoking and heavy episodic alcohol use. Current smoking was defined as smoking cigarettes every day or some days during the week. Heavy episodic alcohol drinking was defined as ≥4 drinks for people assigned female at birth and ≥5 drinks for people assigned male at birth on at least one occasion within the past 30 days.

### 2.3 Independent Variable

The main independent variable in this study, the state policy *Z*‐score, was derived from policy tallies recorded by the Movement Advancement Project (MAP) LGBTQ advocacy group.^55^ MAP tracks over 40 different LGBTQ related laws and policies by state in several major categories (e.g., health care and criminal justice). Each anti‐LGBTQ law detracts one point from the tally, and each pro‐LGBTQ law increases the tally by one point. When a policy does not impact the entire state, fractions of a point are applied.

**FIGURE 1:**
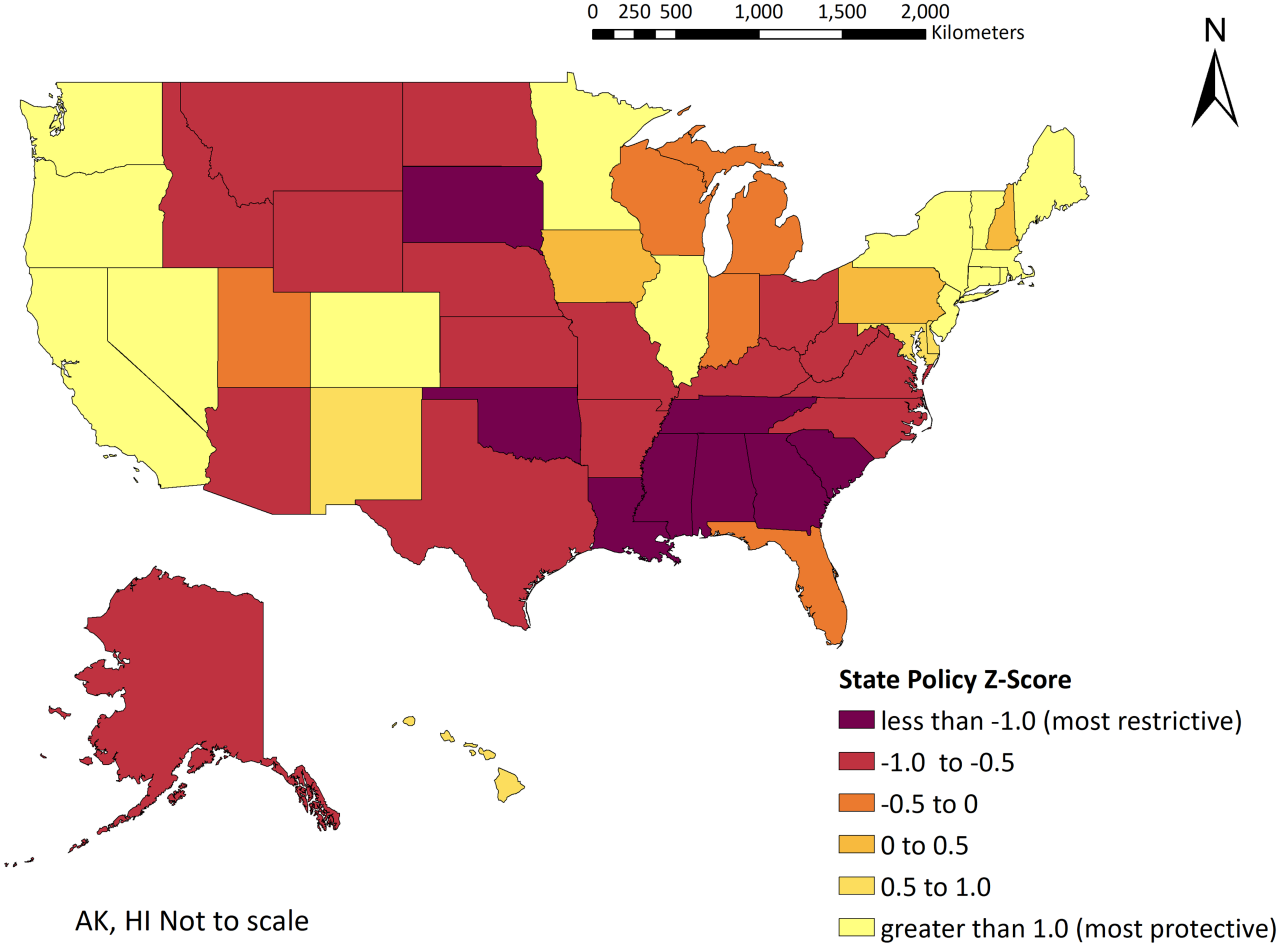
National distribution of state policy *Z*‐Scores for years 2017–2021. AK, Alaska; HI, Hawaii.

Since MAP keeps a real‐time tally of these policies, web archives were used to determine the first policy tally update in January of the corresponding BRFSS year from 2017 to 2021. These tallies were averaged across all 5 years and standardized by converting to a *Z*‐score to address differing denominators by year and to help with interpretability (Table S1). A state policy *Z*‐score of 1 means a state's averaged MAP policy tally is one standard deviation above the mean for all states, and the positive direction indicates a more protective SGM policy environment than the US average. In bivariate analysis states were characterized as restrictive if they scored below the national mean on the policy tally and protective if they scored at or above the national mean. Figure 1 shows the national distribution of state policy *Z*‐scores.

### 2.4 Control variables

We controlled for sociodemographic characteristics known to be associated with cancer and standardly included in SGM cancer research (race/ethnicity, age, sex, educational attainment, household income, employment status, sexual orientation, and gender modality).^70^ Race/ethnicity are social constructs, not biological facts, and the BRFSS categories used in these analyses (American Indian/Alaskan Native, non‐Hispanic; Asian, non‐Hispanic; Black, non‐Hispanic; Hispanic; white, non‐Hispanic) were considered measurable proxies for the impact of systemic and interpersonal racism on health outcomes.^71^ We also controlled for poor access to health care because it is associated with cancer diagnosis and quality of life.^72^, ^73^ We used three dichotomous (yes/no) measures: (1) not having health insurance, (2) avoiding medical care because of costs, and (3) not having a routine checkup in the past year. Lastly, for analyses of cancer survivors, we additionally controlled for cancer types using the two available dichotomous (yes/no) measures: (1) ever told that you have skin cancer? and (2) ever told you had any other types of cancer?

### 2.5 Statistical analyses

Analyses were conducted using R statistical software version 4.2.2. We included weights that BRFSS provides to adjust for the probability of selection and non‐response. Standard errors were adjusted according to CDC guidance.^74^ Because the data were pooled over years, we followed CDC guidance and divided the weights by the proportion of respondents in each year to arrive at an annualized weight.^74^ We utilized the survey statistical package to handle complex survey design and perform weighted analysis for our pooled data.^75^ The study sample was characterized using descriptive statistics and bivariate differences in policy were assessed through chi‐square tests. Multivariate analyses were performed through logistic regression for each of the dichotomous dependent variables. We computed variance inflation factors for all model predictors, which were all 1.5 or less, indicating minimal risk of multicollinearity.^76^ An alpha of 0.05 was set for all statistical tests to determine significance.

## 3 RESULTS

Table 1 displays the sample characteristics stratified by restrictive versus protective state policy *Z*‐scores. The sample was predominantly white, and individuals assigned female at birth comprised a majority. There was an even distribution of ages in the sample. About 9 percent reported being transgender, and almost 50 percent reported being bisexual.

**TABLE 1:** Characteristics of sexual and gender and minoritized population by state policies.

Sample characteristics	Total sample (*N* = 56,309), *n* (%)	Restrictive states (*Z*‐score <0) (*N* = 26,305), *n* (%)	Protective states (*Z*‐Score ≥0) (*N* = 30,004), *n* (%)	*p*‐value
Gender modality				0.015*
Transgender, male‐to‐female	1652 (3.0)	846 (3.1)	806 (2.8)	
Transgender, female‐to‐male	1794 (3.4)	1064 (3.9)	730 (2.8)	
Transgender, gender nonconforming	1206 (2.4)	571 (2.2)	635 (2.6)	
Cisgender	51,657 (91.2)	23,824 (90.7)	27,833 (91.8)	
Sex (assigned at birth)				0.088
Male	24,178 (42.1)	11,117 (41.4)	13,061 (42.9)	
Female	32,131 (57.9)	15,188 (58.6)	16,943 (57.1)	
Sexual orientation				<0.001*
Gay or lesbian	18,106 (29.5)	7855 (28.5)	10,251 (30.7)	
Bisexual	24,306 (46.9)	11,527 (47.3)	12,779 (46.4)	
Straight	2531 (4.1)	1505 (5.0)	1026 (3.1)	
Something else	11,366 (19.5)	5418 (19.2)	5948 (19.8)	
Race				<0.001*
American Indian/Alaska Native	1048 (1.2)	658 (1.5)	390 (0.9)	
Asian	1602 (4.8)	351 (2.7)	1251 (7.2)	
Black	4277 (12.1)	2538 (14.6)	1739 (9.2)	
Hispanic	6171 (18.7)	2506 (17.5)	3665 (20.1)	
White	40,028 (60.0)	19,072 (60.8)	20,956 (59.0)	
Something else	3183 (3.2)	1180 (2.9)	2003 (3.6)	
Age				0.010*
18–24	9218 (27.3)	4594 (28.3)	4624 (26.1)	
25–34	11,496 (25.5)	5448 (24.9)	6048 (26.3)	
35–44	8078 (14.5)	3712 (14.4)	4366 (14.6)	
45–54	7146 (11.1)	3143 (11.4)	4003 (10.8)	
55–64	8629 (10.8)	3780 (10.3)	4849 (11.4)	
65 or older	11,742 (10.7)	5628 (10.7)	6114 (10.7)	
Education level				<0.001*
Did not graduate high school	4131 (13.2)	2159 (14.1)	1972 (12.2)	
Graduated high school	14,142 (27.5)	7259 (29.5)	6883 (25.2)	
Attended college/technical school	16,302 (34.0)	8092 (34.9)	8210 (33.1)	
Graduated college/technical school	21,734 (25.3)	8795 (21.6)	12,939 (29.6)	
Income				<0.001*
Less than $10,000	2796 (5.5)	1444 (5.8)	1352 (5.1)	
$10,000–$14,999	2631 (4.8)	1349 (5.2)	1282 (4.3)	
$15,000–$19,999	3969 (7.2)	2048 (7.7)	1921 (6.6)	
$20,000–$24,999	4706 (8.8)	2417 (9.9)	2289 (7.6)	
$25,000–$34,999	5881 (10.3)	2993 (11.1)	2888 (9.5)	
$35,000–$49,999	6772 (10.6)	3368 (11.4)	3404 (9.7)	
$50,000–$74,999	7268 (11.7)	3363 (11.8)	3905 (11.5)	
$75,000 or more	13,921 (23.8)	5348 (19.8)	8573 (28.4)	
Don't know/refused/missing	8365 (17.4)	3975 (17.3)	4390 (17.4)	
Employment status				0.022*
Unemployed	4226 (9.1)	1852 (9.8)	2374 (8.6)	
Healthcare access measures				<0.001* <0.001* 0.025*
No health insurance	5902 (14.9)	3516 (18.9)	2386 (10.4)	
Did not see medical doctor because of cost	9397 (19.9)	5209 (23.4)	4188 (16.0)	
No routine checkup within past year	14,165 (28.9)	6818 (29.8)	7347 (27.9)	
Cancer prevalence measures				0.002* 0.047* 0.102
Ever diagnosed with cancer	7168 (8.3)	3526 (8.9)	3827 (7.7)	
Ever diagnosed with skin cancer	3627 (3.8)	1768 (4.1)	1859 (3.5)	
Ever diagnosed with other type of cancer	4351 (5.4)	2041 (5.7)	2310 (5.1)	

*Note*: Sample sizes are unweighted and percentages are weighted.

* *p* < 0.05.

SGM people in protective states had higher levels of income (*p* < 0.001), higher levels of education (*p* < 0.001), higher rates of employment (*p* < 0.05), and a higher percentage of respondents in those states were Asian or Hispanic (*p* < 0.001). Restrictive states had a higher proportion of transgender respondents (*p* < 0.05) and a higher proportion of Black, and American Indian or Alaska Native respondents (*p* < 0.05). Restrictive states also had a higher proportion of SGM people who were uninsured (*p* < 0.001), people who avoided seeing a doctor due to cost (*p* < 0.001), and people who did not get a routine checkup in the past year (*p* < 0.05). A greater proportion of SGM individuals living in restrictive states had been previously diagnosed with cancer at the time of the survey (8.9% vs. 7.7%, *p* < 0.01).

Logistic regressions were computed to determine whether the state policy environment was associated with likelihood of a cancer diagnosis in the total analytic sample and outcomes among a subset of cancer survivors (Table 2). After controlling for all sociodemographic variables, SGM individuals diagnosed with cancer had significantly lower odds of living in states with more protective policies (adjusted odds ratio [AOR]: 0.92; 95% confidence interval [CI]: 0.87–0.97). In these analyses, the continuous variable for policy climate was a *Z*‐score ranging from −1.28 to 1.79 (with higher scores indicating more protective policy). Therefore, for example, a one‐unit increase in the state policy *Z*‐score was associated with 8 percent lower odds of an SGM individual being diagnosed with cancer. An increase in state policy *Z*‐score from the most restrictive state (−1.28) to most protective state (1.79) was associated with 25.5 percent lower odds of a cancer diagnosis.

**TABLE 2:** Adjusted logistic regression of likelihood of cancer diagnosis and cancer survivorship outcomes predicted by more protective state LGBTQ policy environments.

Outcome variables	Adjusted odds ratios (95% CI)	Percent change in odds by policy score (−1.28 to 1.79) from most restrictive to most protective state (95% CI)
Total analytic sample (*N* = 56,309)		
Cancer diagnosis	0.92 (0.87, 0.97)*	−25.5 (−34.8, −8.9)*
SGM cancer survivors (*N* = 7168)		
Poor mental health for ≥14 days	0.96 (0.85, 1.08)	−11.8 (−39.3, 26.7)
Depression	0.98 (0.89, 1.09)	−6.0 (−30.1, 30.2)
Current smoking	0.94 (0.83, 1.06)	−17.3 (−43.6, 19.6)
Heavy episodic alcohol use	1.03 (0.89, 1.20)	9.5 (−30.1, 75.0)
Difficulty concentrating or remembering	0.87 (0.78, 0.98)*	−34.8 (−53.4, −6.0)*
Poor physical health for ≥14 days	0.83 (0.74, 0.94)*	−43.6 (−60.3, −17.3)*
Diabetes	0.94 (0.83, 1.06)	−17.3 (−43.6, 19.6)
Cardiovascular disease	0.89 (0.78, 1.01)	−30.1 (−53.4, 3.1)
Serious difficulty walking or climbing stairs	0.90 (0.80, 1.00)*	−27.6 (−49.6, 0)*

*Note*: Odds ratio is the odds of experiencing the outcome associated with a one‐unit change in the state policy *Z*‐score.

Abbreviations: CI, Confidence Interval; LGBTQ, Lesbian, gay, bisexual, transgender, queer; SGM, sexual and gender minoritized.

* *p* < 0.05.

After adjusting for previously described confounders, SGM cancer survivors had significantly lower odds of living in states with protective policies if they had poor physical health (AOR: 0.83; 95% CI: 0.74–0.94), difficulty concentrating or remembering (AOR: 0.87; 95% CI: 0.78–0.98), and/or serious difficulty walking or climbing stairs (AOR: 0.90; 95% CI: 0.80–1.00). An increase in state policy *Z*‐score from most restrictive state (−1.28) to most protective state (1.79) thus reflected 43.6 percent lower odds of poor physical health, 34.8 percent lower odds of difficulty concentrating or remembering, and 27.6 percent lower odds of serious difficulty walking, or climbing stairs. SGM cancer survivors with cardiovascular disease showed a trend (*p* = 0.071) toward lower odds of living in a state with protective policies (AOR: 0.89; 95% CI: 0.78–1.01). We did not find sufficient evidence of an association between state policy and poor mental health, depression, smoking, heavy episodic alcohol use, diabetes, or cardiovascular disease.

## 4 DISCUSSION

Our findings demonstrate that SGM people with a cancer diagnosis are significantly more likely to live in states with restrictive LGBTQ policies after adjusting for sociodemographic characteristics and health access. SGM cancer survivors with poor physical health, and physical and cognitive disabilities are also significantly more likely to live in restrictive policy states. We estimated sizeable decreases in the odds of these outcomes given a one‐unit increase in protective state LGBTQ policy: cancer diagnosis (8%), poor physical health (17%), difficulty concentrating or remembering (13%), and serious difficulty walking or climbing stairs (10%). A policy change from the most restrictive state (*Z*‐score‐1.28) to most protective state (*Z*‐score 1.79) corresponded to substantial decreases in the odds of cancer diagnosis (25.5%), poor physical health (43.6%), cognitive difficulty (34.8%), and difficulty with walking or stairs (27.6%). These results are the first evidence of an association between LGBTQ state polices and SGM cancer prevalence and survivorship outcomes and thus have significant implications for SGM cancer control and future SGM cancer research.

Little is known about how SGM cancer prevalence varies across the United States. Most cancer registries, including the Surveillance, Epidemiology, and End Results (SEER) program, do not record SOGI,^11^, ^77^ and oncology clinics have been slow to operationalize SOGI collection.^78^ This is a major barrier to assessing SGM cancer frequency, distribution, and survival across SGM subpopulations and by specific cancer types nationally. Population health surveys like BRFSS are useful but limited in assessing important clinical factors such as specific cancer diagnosis, stage, and time since diagnosis, which are necessary information to test if LGBTQ policies have a causal relationship with the SGM cancer burden. Future studies should aim to make this contribution, given our evidence that SGM people diagnosed with cancer are more likely to live in restrictive policy states.

Our results indicate that SGM cancer survivors with the most complex medical needs are also more likely to live in restrictive policy states. Regular physical activity is recommended for cancer survivors,^69^ but those with poor physical health and physical disability, more common in restrictive states, are likely less able to engage in it. The same is true for survivors experiencing cognitive difficulty or impairment, which is a common consequence of cancer treatments, particularly chemotherapy.^79^ Poor physical health and cognitive impairment can also limit treatment options, decreasing cancer survival. We did not find significant variation in survivors' mental health or substance use across policy environments, despite these being key SGM health disparities linked to LGBTQ policy in non‐cancer studies.^56^, ^57^, ^58^, ^59^, ^60^ Further studies should clarify this discrepancy. Neither did we detect significant variation by policy in diabetes or cardiovascular disease, although these are important conditions to manage for cancer survivors that disproportionately affect SGM people.^52^, ^80^, ^81^, ^82^, ^83^ It remains unclear why SGM survivors in restrictive states have worse physical health and cognitive disability. Several factors could be behind it: variations in the distribution of cancer types, severity, or length of survivorship, differences in the quality of treatment received, disparities in social support and caregiving, or some unconsidered reason. The mechanisms by which LGBTQ policy could explain these differences should be investigated. It is especially troubling that SGM cancer survivors are more likely to live in restrictive states, and with worse health, given that clinicians and providers are often ill‐equipped to provide culturally tailored care to SGM people.^37^, ^38^, ^40^, ^42^ Protective state LGBTQ policies are important for ensuring access to high‐quality healthcare, training an affirming health care workforce, and reducing stigma for SGM people with cancer.^84^

It is the mandate of state and federal policymakers to improve the collection of SOGI as standard demographics in cancer registries, oncology clinics, and populations health surveillance, following current recommendations,^13^ to assist researchers in understanding how to alleviate the SGM cancer burden. Such data are necessary to explore the mechanisms by which LGBTQ policies may shape or contribute to the SGM cancer burden, which is warranted by the results presented herein. Policy interventions have been effective in reducing SGM health disparities in other domains,^85^ and may be effective levers to advance SGM cancer equity.

Taken together, the results of this study compel further structural analyses in the context of SGM cancer control. Prior studies have largely focused on individual behaviors (e.g., smoking) to explain cancer burden in SGM communities. By neglecting structural discrimination against SGM communities in our research questions and methods, we may fail to address what could be a fundamental cause of SGM cancer inequities.^66^, ^67^ Previous studies have been limited in assessing LGBTQ policy effects because MAP is a real‐time policy tracker,^56^, ^59^, ^62^, ^86^ but this study demonstrated novel use of web archives to assess MAP policy tallies across years. Future studies should consider the following:

1. Study designs should explore the associations between specific policies, or social domains of policy, and the SGM cancer burden. Testing interactions between state policies and individual‐level variables (e.g., health access) could provide deeper insights into the mechanisms driving SGM cancer disparities.
2. Longitudinal studies that follow SGM cancer incidence and survivorship outcomes post‐diagnosis will be necessary to test causal relationships between policy and SGM cancer outcomes. Researchers can examine trends in state policies, whether they remain stable or become more or less restrictive, and explore changes in outcomes before and after the implementation of protective or restrictive policies.
3. SGM subgroups and specific cancer types may be uniquely influenced by policy and should be considered in isolation and with comparative studies.
4. Alternative measures of structural stigma against SGM people^87^ and structural intersectional approaches (e.g., interactions with structural racism)^88^, ^89^, ^90^ should be applied and developed for SGM cancer research.

### 4.1 Limitations

Our study has limitations. First, there is the potential for other state‐level factors to confound the significant associations between LGBTQ state policies and SGM cancer outcomes. For example, states with more restrictive policies have higher poverty rates and tend to more rural geographies, which may shape the allocation of state resources and health services to the general state population. Second, the optional SOGI module was only delivered by 42 states throughout the study period. Missing states were more likely to have restrictive policy scores, meaning the sample was biased toward including SGM people in more protective policy states. Third, in order to power a state‐level analysis, this study aggregated all SGM people which likely masked important subgroup differences. A fourth limitation is that SGM individuals may be reluctant to disclose their identities in population surveys, leading to underreporting.^91^ However, this bias is unlikely to significantly affect our outcomes of interest. Lastly, there was a survivor bias in our interpretations, and the specific diagnosis, time since diagnosis, and treatment history for cancer survivors were unknown, which placed limitations on our interpretation of the relationship between state policies and cancer prevalence and health among survivors.

## 5 CONCLUSION

We found that SGM people diagnosed with cancer are more likely to live in US states with restrictive LGBTQ polices and survivors in these states are more likely to have poor physical health and cognitive disability. These results are timely because record numbers of anti‐LGBTQ bills in recent years have increasingly polarized the LGBTQ policy landscape in the United States.^53^, ^55^ We know that harmful social policies can erode the health and well‐being of SGM communities, though future studies will be needed to understand whether and how these policies shape the SGM cancer burden. Addressing structural discrimination against SGM should become a central focus in cancer research to elucidate how policy reforms may enhance health outcomes. The dismantling of harmful LGBTQ policies and the implementation of protective measures at the local, state, and federal level may be essential steps toward eliminating cancer disparities within SGM communities.

## AUTHOR CONTRIBUTIONS

**Ben C. D. Weideman:** Conceptualization (lead); data curation (lead); formal analysis (lead); investigation (lead); methodology (lead); project administration (lead); software (lead); visualization (lead); writing – original draft (lead). **Donna McAlpine:** Conceptualization (supporting); data curation (supporting); formal analysis (supporting); investigation (supporting); methodology (supporting); project administration (supporting); supervision (lead); validation (supporting); visualization (supporting); writing – review and editing (lead).

## FUNDING INFORMATION

No funding was obtained to support this research.

## CONFLICT OF INTEREST STATEMENT

No competing financial interests exist.

## PRIOR PRESENTATIONS

Presented at the Science of Cancer Health Equity in Sexual and Gender Minority Communities, New York University, New York, NY, October 2023.

## Supporting information


Table S1:

## Data Availability

The data that support the findings of this study are made available by Centers for Disease Control and Prevention, Behavioral Risk Factor Surveillance (https://www.cdc.gov/brfss/index.html) and the Movement Advancement Project (https://www.lgbtmap.org/). These data are available in the public domain.
