# Supplementary material for: State LGBTQ policy environments and the cancer burden in sexual and gender minoritized communities in the United States
Source: Cancer Med. 2024 Aug 14;13(15):e70097. doi: 10.1002/cam4.70097 (PMC11322825; doi:10.1002/cam4.70097)
Supplement: Supplementary file 1 — Table S1: [file CAM4-13-e70097-s001.docx]

## **Supplementary Table S1**: Movement Advancement Project policy tallies by year and calculated state policy Z-score across years

| **State** | **2017** | **2018** | **2019** | **2020** | **2021** | **State Policy**  **Z-score** |
| --- | --- | --- | --- | --- | --- | --- |
| Alabama | 0 | -2.5 | -3.5 | -2 | -6.5 | -1.28 |
| Alaska | 5.5 | 4.5 | 4.25 | 3.75 | 4.25 | -0.67 |
| Arizona | 3.5 | 4 | 3.75 | 3.75 | 5.25 | -0.71 |
| Arkansas | 2.5 | 0.5 | 2 | 1.5 | -0.5 | -0.94 |
| California | 33.5 | 32.5 | 34 | 36.5 | 34.75 | 1.79 |
| Colorado | 23.5 | 23.5 | 24.5 | 29.25 | 33.25 | 1.17 |
| Connecticut | 25.5 | 28 | 27.75 | 30.75 | 34.5 | 1.38 |
| Delaware | 16 | 18.25 | 21.5 | 21.5 | 25.25 | 0.65 |
| Florida | 6.5 | 5.75 | 7.5 | 7 | 10.25 | -0.43 |
| Georgia | -0.5 | -1.5 | -1.5 | -1.5 | 0 | -1.12 |
| Hawaii | 19.5 | 20.5 | 25.5 | 27.25 | 29.75 | 0.98 |
| Idaho | 2.5 | 2 | 3.75 | 2.75 | 2.75 | -0.81 |
| Illinois | 25 | 25.5 | 25.75 | 28.5 | 30 | 1.18 |
| Indiana | 6 | 6.5 | 6.5 | 7.5 | 7.5 | -0.48 |
| Iowa | 14 | 13.5 | 13.5 | 14.5 | 17 | 0.16 |
| Kansas | 3 | 3.75 | 2 | 6 | 6.75 | -0.69 |
| Kentucky | 6 | 6 | 6 | 5.5 | 7.75 | -0.52 |
| Louisiana | 1 | 0.75 | 0.75 | -1.25 | -2.5 | -1.06 |
| Maine | 22 | 20 | 21.25 | 27 | 35 | 1.03 |
| Maryland | 22.5 | 23.25 | 25 | 24.75 | 26.75 | 0.98 |
| Massachusetts | 28 | 27 | 28.5 | 32 | 32.5 | 1.40 |
| Michigan | 4 | 4 | 8.25 | 14.5 | 13.5 | -0.32 |
| Minnesota | 23 | 24.75 | 25.5 | 28.25 | 30 | 1.13 |
| Mississippi | 1.5 | 0 | -1 | 1 | -3.5 | -1.08 |
| Missouri | 4 | 3.75 | 3.5 | 1.5 | 1.25 | -0.81 |
| Montana | 4 | 2.75 | 6.75 | 7.75 | 9 | -0.54 |
| Nebraska | 1 | -0.5 | -0.5 | -0.5 | 3.5 | -0.99 |
| Nevada | 19.5 | 23 | 25 | 30.5 | 34.5 | 1.15 |
| New Hampshire | 10.5 | 11.25 | 17 | 18 | 25.25 | 0.31 |
| New Jersey | 24.5 | 27.5 | 28.75 | 29.75 | 32 | 1.32 |
| New Mexico | 18.5 | 18.25 | 19.5 | 23 | 26.5 | 0.71 |
| New York | 25 | 27.25 | 30 | 35 | 34.75 | 1.47 |
| North Carolina | 3.5 | 4.25 | 2.25 | 3.5 | 4.75 | -0.74 |
| North Dakota | 1.5 | -0.25 | -0.75 | -0.25 | 7 | -0.92 |
| Ohio | 3.5 | 1.75 | 3.25 | 1.75 | 3.75 | -0.81 |
| Oklahoma | 1.5 | 0.5 | -1 | 1 | -1 | -1.03 |
| Oregon | 29.5 | 28.75 | 29.75 | 30.75 | 31.5 | 1.44 |
| Pennsylvania | 11 | 11.25 | 14.5 | 15 | 16 | 0.08 |
| Rhode Island | 27 | 30.25 | 30 | 33.25 | 32.75 | 1.49 |
| South Carolina | 1.5 | 0 | -1.5 | 0.5 | -0.5 | -1.04 |
| South Dakota | 1.5 | -0.5 | -1.5 | -2.5 | -2.5 | -1.13 |
| Tennessee | 0.5 | -1.5 | -1.5 | -2.25 | -4 | -1.19 |
| Texas | 4 | 3.5 | 2.5 | 1.5 | -1.5 | -0.87 |
| Utah | 10 | 9.25 | 8.75 | 10.25 | 13.75 | -0.18 |
| Vermont | 28.5 | 27.5 | 27.25 | 32 | 34 | 1.43 |
| Virginia | 3.5 | 2.5 | 1.75 | 2.25 | 18.75 | -0.56 |
| Washington | 28 | 27.5 | 29.25 | 28.75 | 34.25 | 1.40 |
| West Virginia | 3 | 3.5 | 3.5 | 2 | 5.5 | -0.75 |
| Wisconsin | 11.5 | 8.75 | 10 | 13.5 | 15.5 | -0.06 |
| Wyoming | 2 | 0.25 | 0.75 | 1.5 | 1.75 | -0.94 |

Data are from the Movement Advancement Project: Movement Advancement Project. Available from: <https://www.lgbtmap.org> [Last Accessed; January 24, 2024]
